# Case Report: Intra-aortic balloon pump in a patient with refractory cardiogenic shock complicating severe aortic stenosis—enhanced hemodynamic response with low aortic compliance

**DOI:** 10.3389/fcvm.2025.1587383

**Published:** 2025-06-27

**Authors:** Issei Ota, Kenichiro Sawada, Keita Saku, Teruo Noguchi

**Affiliations:** ^1^ Division of Cardiovascular Intensive Care, Department of Cardiovascular Medicine, National Cerebral and Cardiovascular Center, Suita, Osaka, Japan; ^2^ Department of Cardiovascular Dynamics, National Cerebral and Cardiovascular Center, Suita, Osaka, Japan

**Keywords:** intra-aortic balloon pump, cardiogenic shock, older adults, aortic compliance, hemodynamics

## Abstract

Intra-aortic balloon pump (IABP) counterpulsation generates pressure changes that are primarily influenced by aortic compliance, an index of arterial elasticity that varies widely among patients with cardiovascular disease. However, the potential role of aortic compliance in determining IABP efficacy remains poorly understood. We present the case of an 80-year-old man with severe aortic stenosis and pneumonia who was admitted with generalized fatigue and worsening dyspnea. He developed refractory Society for Cardiovascular Angiography & Interventions stage D cardiogenic shock despite multiple vasopressors. In this patient, who had low aortic compliance (0.62–0.81 ml/mmHg), IABP initiation resulted in immediate hemodynamic improvement through enhanced diastolic augmentation and systolic unloading, leading to rapid reversal of both hypoperfusion and pulmonary congestion. Following successful weaning from vasopressors and IABP removal, the patient underwent transcatheter aortic valve replacement without temporary mechanical circulatory support-related complications. The notable hemodynamic improvement observed in this case highlights the potential importance of aortic compliance as a key determinant of IABP efficacy in elderly patients with cardiogenic shock, suggesting that aortic compliance may help optimize patient selection for IABP support.

## Introduction

The clinical role of the intra-aortic balloon pump (IABP) remains controversial. While the IABP-SHOCK II trial showed no survival benefit in patients with acute myocardial infarction-related cardiogenic shock (AMI-CS) (1), recent large-scale studies have shown non-inferior clinical outcomes with IABP compared to percutaneous ventricular assist devices (pVADs) (2–5). Nonetheless, IABP remains the most widely used temporary mechanical circulatory support (tMCS) device in Japan, accounting for 65.3% of cases compared to venoarterial extracorporeal membrane oxygenation (VA-ECMO, 29.6%) and pVADs (5.0%) (6). This discrepancy in clinical outcomes suggests potential heterogeneity in treatment response among patients receiving IABP support.

The hemodynamic benefits of IABP are achieved through counterpulsation, which involves systolic unloading and diastolic augmentation. During systolic unloading, rapid balloon deflation just before systole (QRS-T interval) lowers peak systemic blood pressure, thereby decreasing left ventricular (LV) afterload. During diastolic augmentation, balloon inflation in diastole (T-P interval) enhances systemic diastolic blood pressure and coronary perfusion pressure (CPP). These IABP-induced pressure changes are fundamentally determined by aortic compliance, an index of arterial elastic properties, which can be approximated clinically by dividing stroke volume by pulse pressure. Aortic compliance decreases with age and the progression of cardiovascular disease, although it varies significantly among individuals (7). Prior studies suggest that lower compliance may amplify IABP-induced pressure changes (8, 9).

Despite its critical role in IABP hemodynamics, the influence of aortic compliance on IABP efficacy remains underexplored, with limited supporting clinical evidence. A better understanding of this relationship could explain the heterogeneous treatment responses and help optimize patient selection for IABP support. Here, we present a case that demonstrates the potential importance of aortic compliance in determining IABP effectiveness in an elderly patient with refractory cardiogenic shock.

## Case description

An 80-year-old man with a history of hypertension and chronic kidney disease stage (CKD) 3b presented to the emergency department with generalized fatigue. His initial vital signs included a blood pressure (BP) of 109/49 mmHg and a heart rate (HR) of 77 beats per min. Despite receiving oxygen of 10 L/min via reservoir mask, he remained tachypneic with a respiratory rate of 28 breaths per min and hypoxic with an oxygen saturation of 89%. His body temperature was 36.2°C.

Physical examination revealed bilateral pulmonary crackles and a grade 4/6 systolic ejection murmur at the right second intercostal space. Additional findings included jugular vein distention, mild peripheral edema, and warm extremities. Laboratory results showed metabolic acidosis (pH 7.275, pCO_2_ 31.3 mmHg, HCO_3_ 14.1 mmol/L, lactate 3.3 mmol/L), hemoglobin 11.1 g/dl, white blood cell count of 9,590/µl, c-reactive protein concentration (CRP) of 2.23 mg/dl, and elevated B-type natriuretic peptide (BNP) at 4,301 pg/ml. Electrocardiography demonstrated sinus rhythm with T-wave inversion in the precordial and inferior leads. Chest radiograph showed increased pulmonary vascular markings and right lower lung field infiltration. Echocardiography revealed newly reduced LV ejection fraction (LVEF 30%, down from 60%) and severe aortic stenosis (AS) with a peak velocity of 4.4 m/s, mean pressure gradient of 50 mmHg, and an aortic valve area of 0.7 cm^2^ as calculated using the continuity equation. Subsequent coronary angiography showed no significant coronary artery stenosis. Right heart catheterization showed a pulmonary capillary wedge pressure (PCWP) of 18 mmHg, pulmonary artery pressure (PAP) of 30/20 mmHg, right atrial pressure (RAP) of 7 mmHg, cardiac index (CI) of 1.91 L/min/m^2^ obtained from thermodilution cardiac output, and systemic vascular resistance (SVR) of 1,548 dyne·s/cm^5^. In this case, the estimated aortic compliance range was 0.62–0.81 ml/mmHg, calculated by dividing the stroke volume obtained from thermodilution cardiac output by pulse pressure. Given that the normal range is 1.2–2.4 ml/mmHg (8), these values indicate a substantial reduction in aortic compliance in this patient. During hospitalization, PCWP was estimated from diastolic PAP, which measured 20 mmHg and corresponded with the directly measured PCWP of 18 mmHg (10). Pulmonary vascular resistance (PVR) was calculated to be 133 dyne·s/cm^5^, indicating no significant elevation.

**Figure 1:**
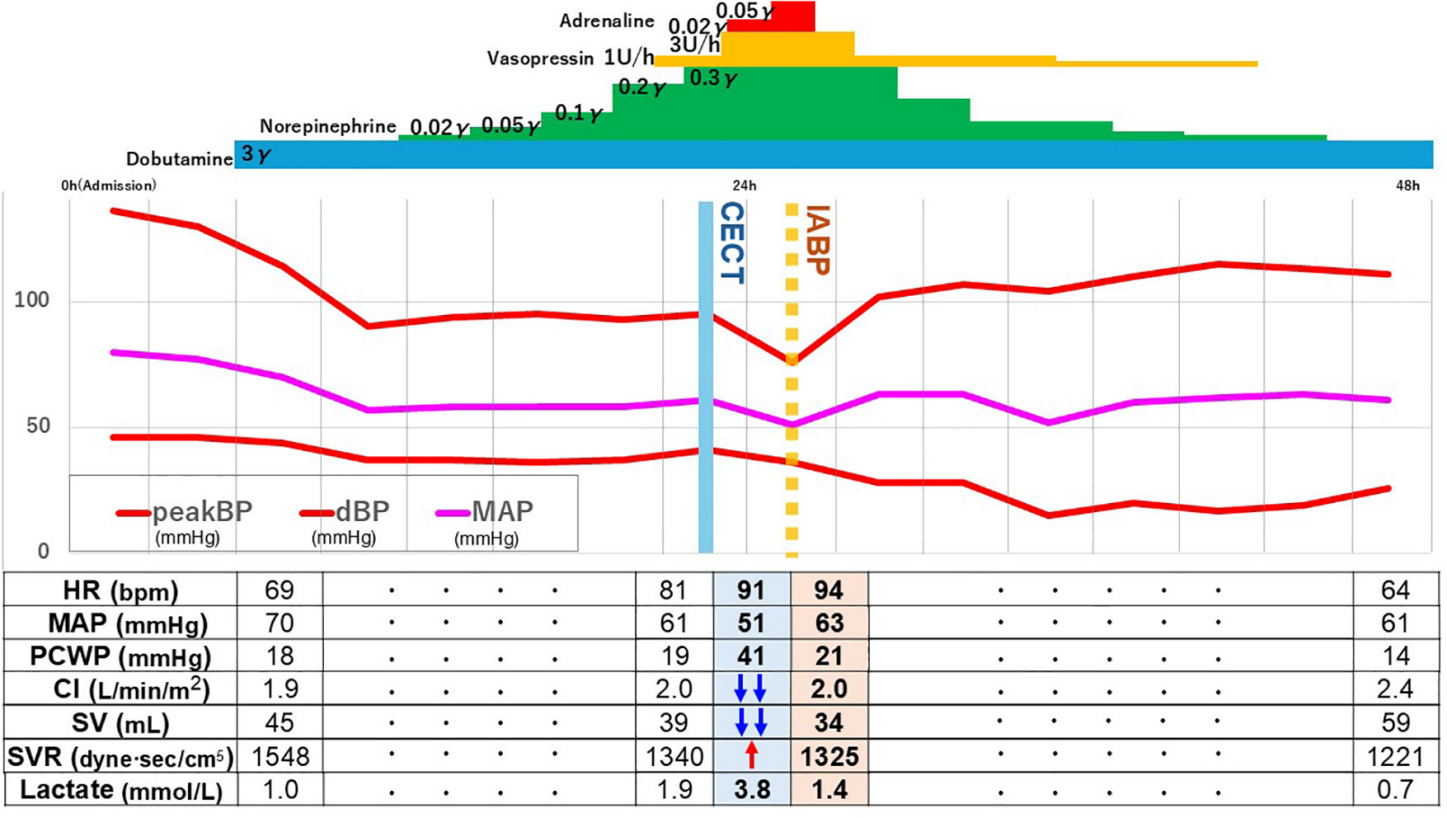
The hemodynamic course over the first 48 h following admission. Following contrast-enhanced computed tomography, further hemodynamic deterioration was observed, necessitating increased catecholamine doses. Directional arrows (↓↓severe reduction, ↑elevation) indicate clinical trajectory based on physical examination and available pressure data. However, significant improvement was achieved with the initiation of IABP. CECT, contrast-enhanced computed tomography; CI, cardiac index; dBP, diastolic blood pressure; HR, heart rate; MAP, mean arterial pressure; PCWP, pulmonary capillary wedge pressure; peakBP, peak blood pressure; SV, stroke volume; SVR, systemic vascular resistance.

The patient was diagnosed with Society for Cardiovascular Angiography and Interventions (SCAI) stage B cardiogenic shock complicated by severe respiratory failure with a PaO_2_/FiO_2_ (P/F) ratio of 76 mmHg due to pulmonary congestion and pneumonia. Initial management included invasive mechanical ventilation, hemodynamic support with dobutamine at 3 µg/kg/min and norepinephrine at 0.02 µg/kg/min, and antimicrobial therapy using ampicillin/sulbactam at 3 grams every 8 h. After 9 h, peripheral hypoperfusion improved (lactate 0.7 mmol/L); however, norepinephrine requirements increased, and elevated PCWP and low CI persisted. Following a heart team conference to discuss definitive treatment for severe AS, contrast-enhanced computed tomography was performed to assess candidacy for transcatheter aortic valve replacement (TAVR). Following the computer tomography scan, the patient developed progressive hypotension and peripheral hypoperfusion. Despite escalating vasopressor support (dobutamine at 3 µg/kg/min, norepinephrine at 0.2 µg/kg/min, vasopressin at 3 U/h, and epinephrine at 0.05 µg/kg/min), mean arterial pressure (MAP) remained at 51 mmHg. Peripheral hypoperfusion worsened (lactate increased from 0.7 to 3.8 mmol/L) and severe pulmonary congestion developed (P/F ratio declined from 186 to 84; PCWP increased from 16 to 41 mmHg) (Figure 1).

**Figure 2:**
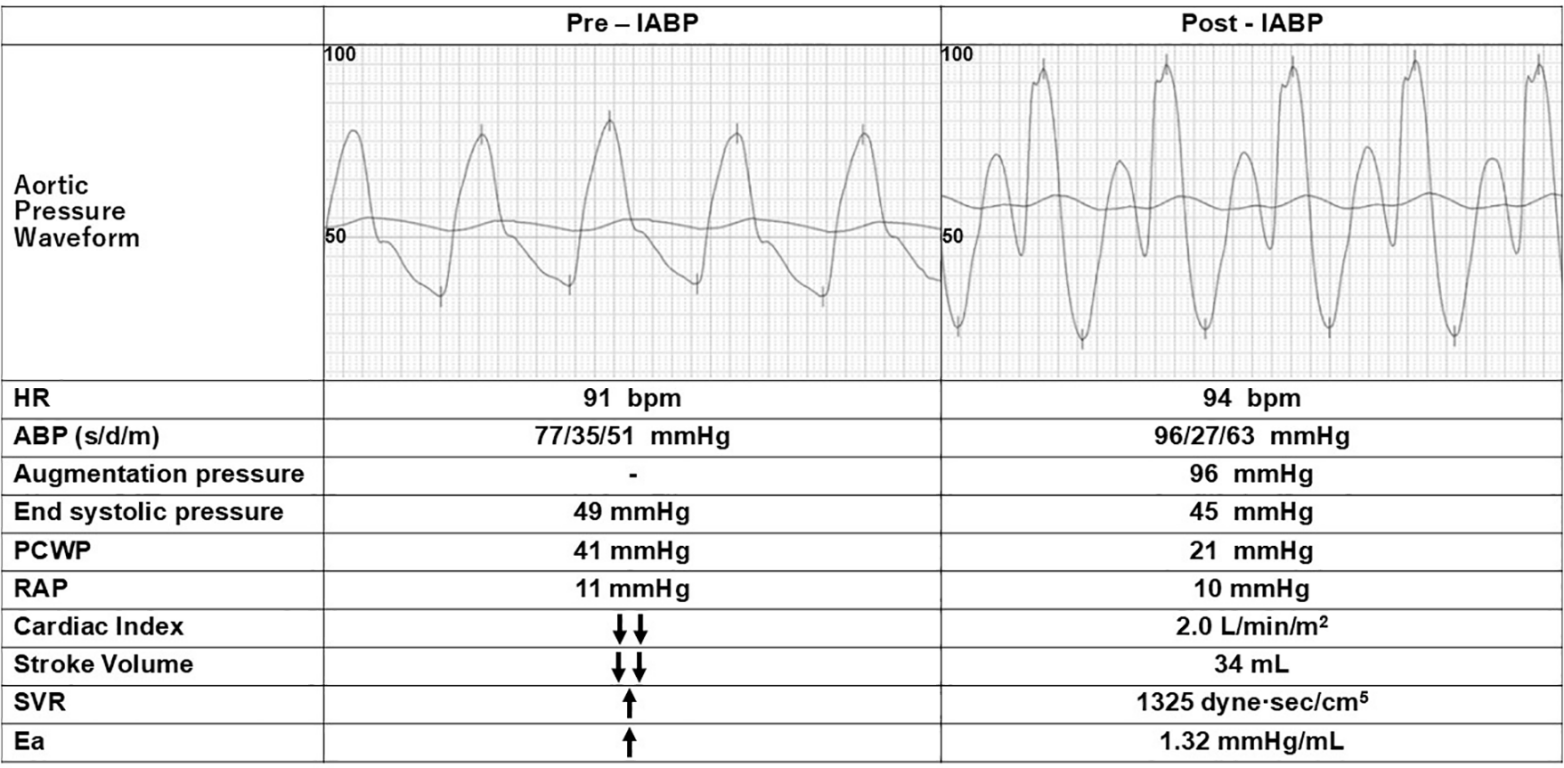
Hemodynamic changes before and after IABP initiation. The initiation of IABP resulted in a rapid increase in blood pressure and a significant reduction in PCWP. The middle line in the aortic pressure waveform represents mean arterial pressure. Directional arrows are as described in Figure 1. ABP (s/d/m), arterial blood pressure (systolic/diastolic/mean); Ea, effective arterial elastance; HR, heart rate; PCWP, pulmonary capillary wedge pressure; RAP, right atrial pressure; SVR, systemic vascular resistance.

Given the refractory wet-cold profile based on the Nohria-Stevenson classification and SCAI stage D, tMCS was indicated. Upon arrival in the catheterization laboratory, MAP was stable at 51 mmHg. Considering the patient's advanced age and risk-benefit profile, IABP was selected as the initial support strategy. Intra-aortic balloon pump initiation resulted in immediate hemodynamic improvements, with MAP increasing from 51 to 63 mmHg and end-systolic pressure (ESP) decreasing from 49 to 45 mmHg (Figure 2). Pulmonary capillary wedge pressure decreased substantially from 41 to 21 mmHg. Cardiac index improved to 2.0 L/min/m^2^, comparable to pre-deterioration values, demonstrating effective systolic unloading and diastolic augmentation.

At 18 h after post-IABP initiation, all vasopressors were successfully discontinued. Dobutamine was maintained at 3 µg/kg/min. Hemodynamics showed sustained improvement: MAP of 61 mmHg, HR of 64 bpm, PAP of 25/14 mmHg, right atrial pressure of 3 mmHg, CI of 2.4 L/min/m^2^, and SVR of 1,221 dyne·s/cm^5^ (Figure 1). The IABP was successfully removed after 52 h of support. On hospital day 8, the patient underwent uncomplicated trans-femoral TAVR under strict infection precautions. Following TAVR, dobutamine was weaned, and guideline-directed medical therapy was initiated. A pre-discharge echocardiography revealed improvement in LVEF from 30% to 44%. The patient was discharged to rehabilitation on postoperative day 24 and maintains function mobility during ongoing outpatient follow-up.

## Discussion

We describe an elderly patient with severe AS and pneumonia who developed refractory SCAI stage D cardiogenic shock despite multiple vasopressors. The patient exhibited low aortic compliance (0.62–0.81 ml/mmHg) and demonstrated notable hemodynamic improvement following IABP support. Previous observational studies have characterized potential IABP responders in hypoperfused acute decompensated heart failure as those with elevated SVR, isolated LV or biventricular dysfunction, pulmonary congestion, and absence of excessive tachycardia (11–13). Although our patient exhibited several of these favorable characteristics, importantly, the shock had progressed to SCAI stage D, where IABP is generally considered insufficient (14, 15). Nevertheless, the immediate and substantial improvement in both hypoperfusion and pulmonary congestion led us to consider the role of the patient's low aortic compliance as a potential determinant of IABP efficacy, particularly in older adult patients.

The hemodynamic effects of IABP fundamentally depend on the interaction between balloon inflation/deflation and the mechanical properties of the arterial system (8). Aortic compliance is a major determinant of the degree of systolic unloading and diastolic augmentation. During diastole, the lower the compliance, the greater the increase in diastolic augmentation pressure. This effect contributes to an increase in MAP and CPP, thereby improving peripheral hypoperfusion and coronary blood flow. During systole, the lower the compliance, the greater the decrease in ESP. This, in turn, leads to a greater reduction in LV afterload, which results in an increase in stroke volume and a decrease in PCWP.

Previous studies have demonstrated that lower aortic compliance amplifies the hemodynamic effects of IABP, as pressure changes are determined by the ratio of balloon volume to aortic compliance. Papaioannou et al. demonstrated greater pressure augmentation with decreased compliance *in vitro* (8), and observed similar findings in patients with AMICS (9). Although low aortic compliance is generally considered disadvantageous in cardiovascular disease (16), it may enhance IABP effectiveness throughout the cardiac cycle. While this physiological relationship has been shown, our case uniquely demonstrates its clinical significance in SCAI stage D shock. This observation suggests that aortic compliance assessment might help identify patients who could benefit from IABP support, even in refractory cardiogenic shock.

**Figure 3:**
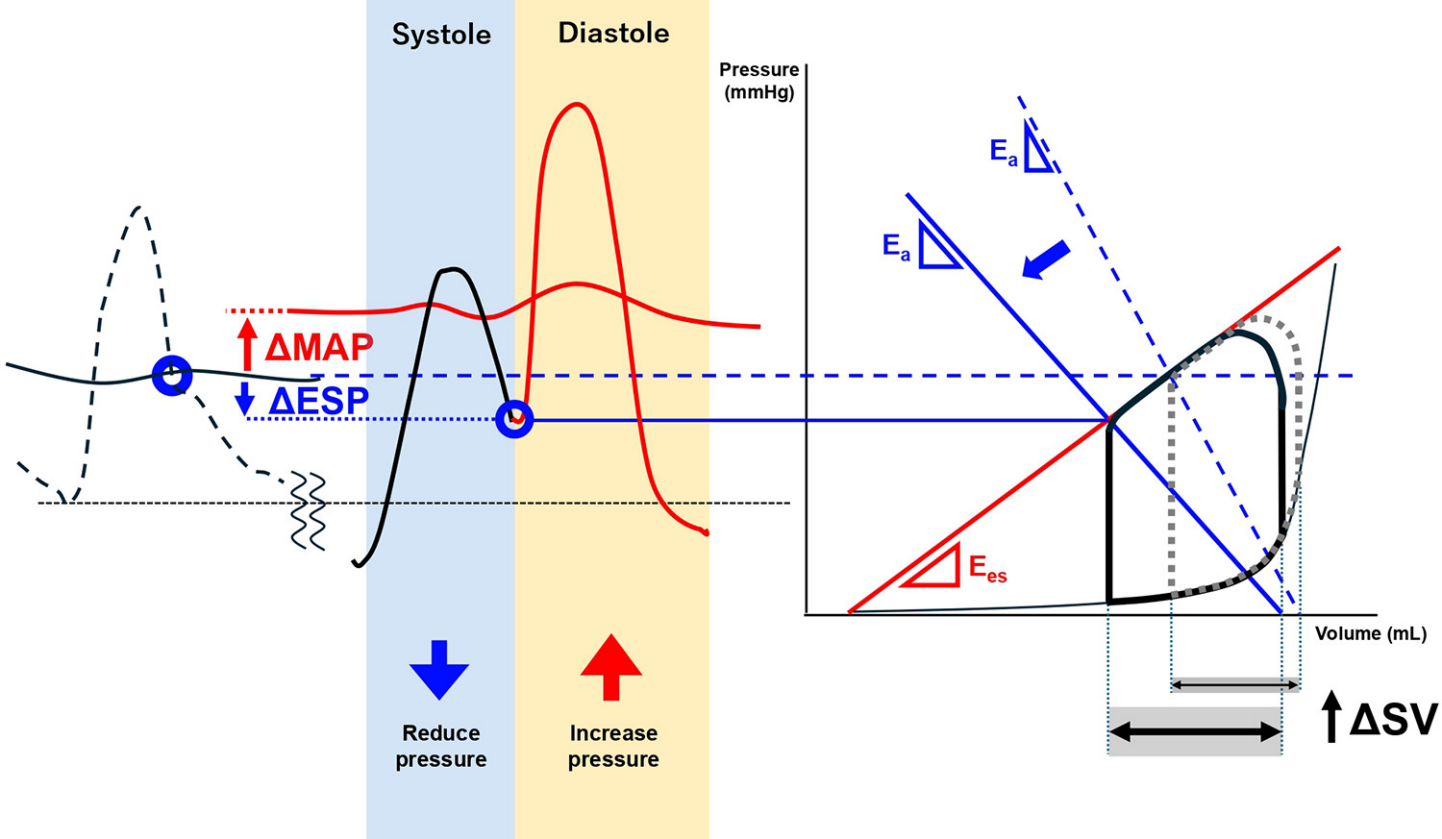
Hemodynamic effects of IABP in A patient with Low aortic compliance. This figure illustrates the hemodynamic changes induced by intra-aortic balloon pump (IABP) support in a patient with low aortic compliance, severe aortic stenosis, and left ventricular dysfunction. The dotted line (…) represents the state before IABP insertion, while the solid line (—) represents the state after IABP insertion. The pressure-dynamic effects of IABP are achieved through balloon deflation just before systole (systolic unloading), which lowers end-systolic pressure (Ea), increases stroke volume, and reduces left ventricular end-diastolic pressure, as shown in the pressure-volume (PV) loop on the right. These effects improve ventriculo-arterial coupling through reduced Ea, which is particularly beneficial in the presence of impaired contractility (Ees). During diastole, balloon inflation (diastolic augmentation) increases augmentation pressure without imposing additional left ventricular afterload, thereby enhancing coronary perfusion pressure and maintaining or increasing mean arterial pressure, as shown in the pressure waveform on the left. The pressure changes become greater as compliance decreases, resulting in enhanced hemodynamic response. Ea, effective arterial elastance; Ees, end-systolic elastance; ESP, end-systolic pressure; MAP, mean arterial pressure; SV, stroke volume. Ea represents left ventricular afterload. Ees represents left ventricular contractility.

In our case, the patient exhibited lower-than-normal aortic compliance (0.62–0.81 ml/mmHg) during the acute phase. Intra-aortic balloon pump support resulted in immediate hemodynamic improvement as evidenced by increased diastolic augmentation without increasing afterload (MAP rising from 51 to 63 mmHg and lactate levels decreasing from 3.8 to 1.4 mmol/L). Additionally, ESP decreased from 49 to 45 mmHg), and pulmonary congestion improved (PCWP reduced from 41 to 21 mmHg). Given the reduced LVEF (30%), this enhanced afterload reduction was particularly effective in improving stroke volume and reducing PCWP, consistent with previously reported benefits of afterload reduction in severe AS with LV dysfunction (17). The benefits of IABP-mediated afterload reduction specifically in patients with severe AS have been previously demonstrated (18). In addition, the enhanced CPP (increased from 35 to 96 mmHg) (19) may have contributed to LV systolic functional recovery through improved coronary perfusion in severe AS with impaired coronary microcirculation (20). Figure 3 shows the hemodynamic effects of IABP in our patient with low aortic compliance.

Sustained hemodynamic improvements were observed at 24 h post-initiation (MAP: 64 mmHg, lactate: 0.7 mmol/L, CI: 2.4 L/min/m^2^, and PCWP: 14 mmHg), facilitating rapid withdrawal of catecholamines and suggesting improved LV function.

Finally, compared to pVADs and VA-ECMO, IABP use has been associated with significantly lower rates of adverse events such as bleeding, stroke, acute kidney injury, limb ischemia, hemolysis, and sepsis, partly due to its smaller sheath size (7.5 Fr to 8 Fr) (21). Given that tMCS-related complications are associated with poor prognosis (21), IABP may be a preferable option when sufficient hemodynamic improvement is anticipated with this less invasive approach, as demonstrated in our case where successful circulatory support was achieved without MCS-related complications. Therefore, this case highlights aortic compliance as a potential key predictor of IABP efficacy, particularly in older adult patients at high risk for tMCS-related complications.

## Limitations

Several important limitations should be noted. First, although we observed significant hemodynamic improvement with IABP, we could not directly evaluate changes in myocardial contractility during the period when the patient was deteriorating. Second, although we observed enhanced CPP following IABP support, we could not definitively attribute the improved LV function to recovery from myocardial ischemia, as there were no ischemic electrocardiogram changes before or after IABP insertion. Third, this is a single case observation, and the relationship between aortic compliance and IABP efficacy requires validation in larger studies including comparisons with other tMCS devices. Fourth, while our findings suggest a relationship between low aortic compliance and an enhanced IABP response, we acknowledge that multiple conditions, including severe AS, reduced LV function, and pneumonia, may have created hemodynamics interactions that prevent attributing the observed IABP benefits solely to aortic compliance. Finally, the potential increased risk of vascular complications such as limb ischemia and peripheral embolization in patients with low aortic compliance needs to be evaluated, as reduced compliance often reflects underlying arterial disease and poor vascular integrity.

## Conclusion

This case demonstrates successful management of an older adult with SCAI stage D cardiogenic shock with IABP support in the context of low aortic compliance. Our findings suggest that aortic compliance might be a key determinant of IABP efficacy and could potentially guide patient selection for this less invasive tMCS approach.

## Data Availability

The original contributions presented in the study are included in the article/Supplementary Material, further inquiries can be directed to the corresponding author.
