# Supplementary material for: Case Report: Intra-aortic balloon pump in a patient with refractory cardiogenic shock complicating severe aortic stenosis—enhanced hemodynamic response with low aortic compliance
Source: Front Cardiovasc Med. 2025 Jun 27;12:1587383. doi: 10.3389/fcvm.2025.1587383 (PMC12245855; doi:10.3389/fcvm.2025.1587383)
Supplement: Supplementary file 1 [file Table1.pdf]

Supplementary Table S1

Laboratory Data

Na	134	mEq/L
K	5.3	mEq/L
Ca	8.6	mEq/L
iP	6.3	mEq/L
BUN	92	mg/dL
CRE	4.23	mg/dL
UA	13.5	mg/dL
CK	65	U/L
CK-MB	9	U/L
CRP	2.23	mg/dL
TP	6.9	g/dL
ALB	2.8	g/dL
T-BIL	0.3	mg/dL
AST	23	U/L
ALT	23	U/L
LD	278	U/L
Glucose	333	mg/dL
LDL	59	mg/dL

WBC	9.59	/uL
HGB	11.1	g/dL
Hct	33.1	
PLT	260	/uL
D-dimer	2.5	μg/mL
TnT	0.25	ng/mL
BNP	4301	pg/mL

pH (Vgas)	7.275	
pO2	46.6	mmHg
pCO2	31.3	mmHg
HCO3	14.1	mmol/L
Lac	3.3	mmol/L
COVID19Ag	(-)	
flu	(-)	
