# Supplementary material for: Case Report: Intra-aortic balloon pump in a patient with refractory cardiogenic shock complicating severe aortic stenosis—enhanced hemodynamic response with low aortic compliance
Source: Front Cardiovasc Med. 2025 Jun 27;12:1587383. doi: 10.3389/fcvm.2025.1587383 (PMC12245855; doi:10.3389/fcvm.2025.1587383)
Supplement: Supplementary file 2 [file Table2.pdf]

**Supplementary Table S2**

**Transthoracic echocardiography data**

| | | |
|---|---|---|
| IVSd/PWd | 8/10 | mm |
| LVDd/Ds | 58/49 | mm |
| LVEF | 25-30 | % |
| LV wall motion | Mid-apical severe hypokinesis | |
| LVOT VTI | 15 | cm |
| TR | Mild | |
| MR | Mild | |
| TRPG | 38 | mmHg |
| E/A | 62/105 | |
| DcT | 175 | ms |
| Vmax | 4.6 | m/s |
| meanPG | 55 | mmHg |
| AVA | 0.702 | $cm^2$ |
| AR | Mild | |
| IVC | 10/24 | mm |
| IVC RC | (+) | |
